# Development and Formative Usability Evaluation of a Theory-Driven Progressive Web Application for Young Adult Wellness Engagement (MiCARE): Protocol for a Mixed Methods Study

**DOI:** 10.2196/86515

**Published:** 2026-03-24

**Authors:** Ayesha Thanthrige, Nilmini Wickramasinghe

**Affiliations:** 1 School of Computing, Engineering and Mathematical Sciences, La Trobe University, Kingsbury Drive, Bundoora, Victoria, 3086, Australia, 61 430 601 237

**Keywords:** wellness engagement, young adults, progressive web application, prediabetes prevention, formative usability evaluation, mixed methods study, self-determination theory, task-technology fit, unified theory of acceptance and use of technology, digital health intervention

## Abstract

**Background:**

Young adults face rising wellness challenges, including prediabetes risk, requiring sustained engagement with preventive health interventions. Digital wellness applications offer promise for promoting healthy lifestyle behaviors, yet high dropout rates and inadequate personalization limit their effectiveness. This paper outlines the technical implementation and formative usability evaluation of MiCARE, a theory-driven progressive web application (PWA) designed to support sustained wellness engagement among young adults through user-centered design.

**Objective:**

This study aims to systematically implement theory-driven design specifications into a functional web application, the MiCARE platform, and to conduct a formative usability evaluation with a convenience sample of 20 university-affiliated young adults aged 18 to 34 years in Victoria, Australia, in both rural and urban areas using the task-technology fit and unified theory of acceptance and use of technology frameworks as organizing lenses to assess usability, usefulness, and satisfaction.

**Methods:**

This is an embedded mixed methods study conducted across 2 phases: phase 3 and phase 4. Phase 3 involves the technical implementation of 6 theory-driven features (ie, empathetic chatbot, learning hub, dynamic goal setting, gamification, personalized reminders, and progress dashboard) using HTML5, CSS3, JavaScript, Google Dialogflow ES, and Firebase services, following the Agile methodology over 6 months with biweekly self-managed sprints and clinical verification. Phase 4 is a 3-month formative usability feasibility evaluation with 20 young adults recruited from La Trobe University (Bundoora and Bendigo campuses). Participants will complete screening and initial, midpoint, and final surveys assessing usability, usefulness, and satisfaction, while real-time use analytics captures engagement patterns. Data analysis will use the task-technology fit and unified theory of acceptance and use of technology frameworks as interpretive guides, with quantitative data analyzed using descriptive statistics (R Studio) and qualitative feedback analyzed through thematic analysis (NVivo). Use analytics will provide descriptive contextual information only. The study has received ethics approval from the La Trobe University Human Research Ethics Committee (HEC24507).

**Results:**

The study will take place between 2025 and 2026. Phase 3 (technical implementation) commenced in October 2025 and is currently ongoing, with core features under active development and verification. Phase 4 (formative usability and feasibility evaluation) is scheduled to commence following completion of phase 3. Evaluation results will be disseminated in academic forums and peer-reviewed publications in early 2027. The findings will enable us to evaluate the feasibility, acceptability, and usability of a theory-driven PWA in this university-affiliated sample, informing refinements and future larger-scale studies.

**Conclusions:**

This study will contribute to the technical implementation and formative usability evaluation of a multitheoretical, user-centered PWA for wellness engagement in preventive health, bridging the gap between conceptual frameworks and deployed interventions.

## Introduction

### Background

Over recent years, mobile health (mHealth) technologies have become integral to contemporary wellness promotion, with more than 350,000 health applications available globally [1]. Wellness applications represent prevalent mHealth tools designed to promote healthy lifestyle behaviors, such as improved diet and physical activity [2]. In Australia, approximately 88% of the population owns a smartphone [3], offering significant opportunities for large-scale digital health interventions.

Despite this proliferation, sustained engagement remains a critical challenge. Meta-analyses report dropout rates exceeding 43% across mHealth interventions for chronic disease management [4]. Among young adults aged 18 to 34 years, defined by the Australian Communications and Media Authority as digital natives [5], engagement barriers are particularly pronounced due to inadequate personalization [6], cultural insensitivity [7], and a lack of theoretical grounding in application design [8]. Building on the completed phase 1 (systematic literature review of 32 studies, expert consultations [n=6], formative research with young adults [n=20], and rapid review of user interface [UI] or user experience [UX] design practices [35 studies]) and phase 2 (wireframe development and preliminary evaluation using interactive Figma prototypes refined through iterative expert feedback), the MiCARE progressive web application (PWA) aims to address these gaps through a theory-driven, user-centered approach.

Young adults face rising chronic disease risks, particularly prediabetes, which affects approximately 374 million adults globally [9] and can be reversed through early lifestyle intervention [10]. Sedentary behavior compounds these risks, with adults spending an average of 8.8 hours per day sedentary across European countries [11], while Australians sit for approximately 8 hours per day [12]. Poor dietary habits further exacerbate health risks, with nearly half of Australian adults (49%) failing to consume recommended fruit servings and more than 90% not meeting vegetable intake guidelines [13].

Despite these challenges, digital wellness solutions offer unique advantages for young adults, including flexible access to tailored tools and seamless integration into daily routines [14]. However, most commercially available applications lack evidence-based design and theoretical grounding [8,15], compromising personalization and cultural relevance and resulting in poor sustained engagement.

### Theoretical Foundation and Prior Development

The MiCARE framework synthesizes multitheoretical models to address engagement gaps: self-determination theory (SDT) [16], which emphasizes autonomy, competence, and relatedness as core constructs underpinning intrinsic motivation; the CARE (compassion, assistance, respect, and empathy) framework [17], which guides culturally sensitive engagement through compassion, assistance, respect, and empathy; user-centered design (UCD) principles [18], which emphasize iterative co-design and responsiveness to user preferences; inclusive design principles [19], which ensure equitable access and representation for diverse populations; design science research methodology (DSRM) [20,21], which provides a systematic approach to developing and evaluating information systems artifacts; and task-technology fit (TTF) [22] and Unified Theory of Acceptance and Use of Technology (UTAUT) [23], frameworks for assessing technology acceptance and sustained use.

Prior systematic work established the foundation for MiCARE development. A systematic literature review of 32 studies identified key engagement barriers, including high dropout rates [24,25], inadequate personalization [26,27], environmental constraints [7,28], and cultural or language barriers [29,30]. Facilitators included cultural tailoring [29,30], personalized feedback [25,31], user-friendly design [32,33], and peer support [34,35].

Building on these findings, a 3-step UCD process included systematic literature review (32 studies), expert consultations (n=6), formative research with young adults (n=20), and rapid review of UI and UX design practices (35 studies). This process generated 5 design objectives: empathy-driven interaction, equity-focused accessibility, culturally sensitive personalization, incremental goal setting, and intuitive onboarding. User preferences identified during earlier formative design research with young adults indicated that 55% preferred grid layouts, 65% favored large-button interactions, and 50% preferred minimalist interface designs, informing the feature specifications.

### The Implementation and Evaluation Gap

While conceptual frameworks for digital health interventions are well established [36,37], the technical translation from theory to deployable software constitutes a critical knowledge gap [38]. Published studies typically present either fully developed interventions without implementation details or propose frameworks without functional prototypes. For example, LeSeure et al [29] developed the DiaFriend mobile app for type 2 diabetes management but noted that “the backend is incomplete and requires expert collaboration for full functionality.” Similarly, Curtis et al [39] documented UCD processes for a childhood weight management app but provided limited technical specifications, constraining reproducibility. This implementation gap is particularly problematic for resource-constrained research settings. Comprehensive technical documentation enables future researchers to adapt proven implementations rather than rebuilding from conceptual frameworks, thereby accelerating evidence-based innovation in digital health [40].

Furthermore, rigorous evaluation of theory-driven wellness applications remains limited. While the TTF [22] and UTAUT [23] provide validated frameworks for assessing technology adoption, few studies systematically apply these models to evaluate user experience outcomes in preventive health contexts [38]. The integration of multiple theoretical perspectives (SDT, CARE, UCD, and inclusive design) with evaluation frameworks (TTF and UTAUT) addresses calls for theory-driven development [36,37] and rigorous evaluation methodologies [40] in digital health.

### Study Aims

This paper presents a protocol for a 9-month project with the following aims:

- Phase 3 (months 1‐6)—systematically implement theory-driven design specifications into a functional PWA, documenting the technical translation process from design rationale to deployable code
- Phase 4 (months 7‐9)—conduct a formative usability, acceptability, and feasibility evaluation with a convenience sample of university-affiliated young adults from La Trobe University, using the TTF and UTAUT frameworks as organizing lenses to assess usability, usefulness, and satisfaction

We hypothesize that a theory-driven, user-centered digital health platform will demonstrate high usability, perceived usefulness, and user satisfaction among young adults, supporting sustained engagement with wellness content over a 3-month evaluation period. This study assesses formative usability and feasibility to identify design strengths, usability issues, and refinement priorities, informing subsequent development and larger-scale evaluation.

## Methods

### Overview

The development and evaluation of the platform adopt a co-design, user-centric methodological approach [40], weighing all stakeholders’ experiential needs and preferences equally. The implementation process follows the DSRM [20,21], integrating behavioral theory (SDT and CARE framework) with UCD principles [18] and inclusive design [19] to optimize uptake and use of the digital platform.

Integrating digital health technologies into practice has the potential to impact current practices [41]. The design process is guided by multiple theoretical frameworks, including SDT for motivation and autonomy, CARE for empathetic engagement, UCD for iterative co-design, and inclusive design for accessibility, ensuring theoretical fidelity throughout development. The evaluation uses the TTF [22] and the UTAUT [23] frameworks to match stakeholder expectations and digital literacy to the final platform design. The evaluation follows established usability testing guidelines [42], with sample sizes appropriate for identifying usability issues and achieving qualitative saturation [43].

### Study Design

**Figure 1.**
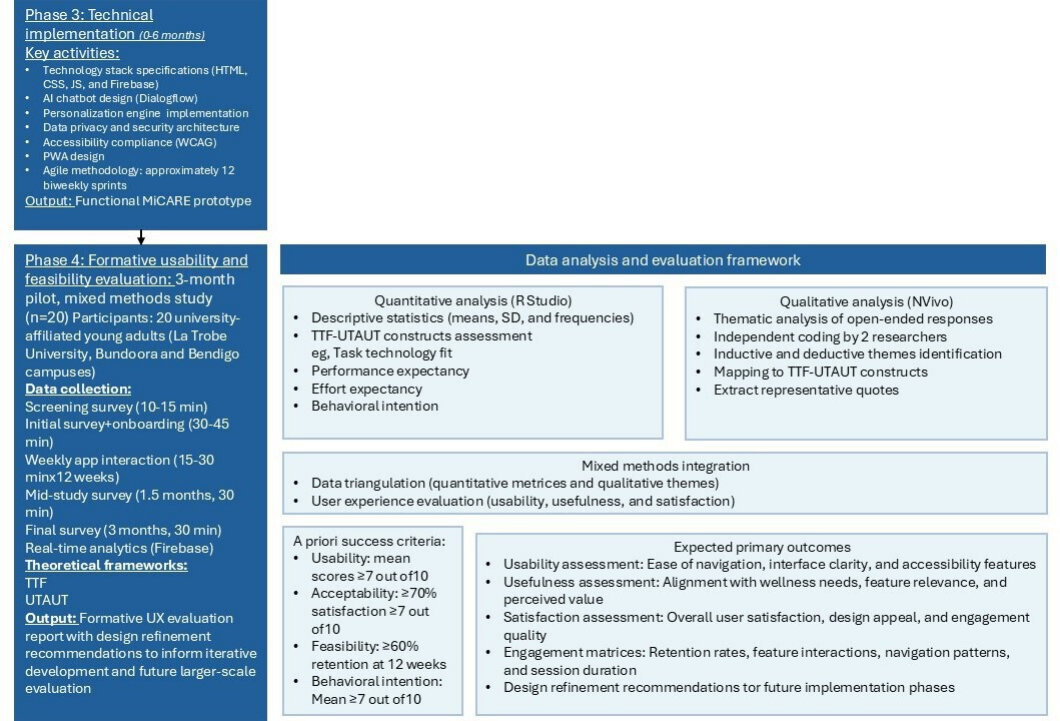
Overview of phase 3 implementation and phase 4 evaluation process. AI: artificial intelligence; PWA: progressive web application; TTF: task-technology fit; UTAUT: unified theory of acceptance and use of technology; UX: user experience; WCAG: Web Content Accessibility Guidelines.

The study uses a mixed methods approach across 2 phases conducted over 9 months. An overview of the study design is shown in Figure 1. Phase 1 [44] (systematic literature review, expert consultations, formative research, and UI and UX review) and phase 2 (wireframe development and prototype evaluation, which has been accepted for publication) have been completed, providing the theoretical and design foundation for the subsequent phases. Phase 3 (October 2025 to March 2026) has commenced and is currently underway, involving the technical implementation of the MiCARE platform. Phase 4 (April 2026 to June 2026) will follow the completion of phase 3 and will focus on formative usability and feasibility evaluation with 20 university-affiliated young adults from La Trobe University (Bundoora and Bendigo campuses). This single-site, convenience sample supports heuristic usability discovery and design validation.

### Participants and Setting

The stakeholder group for this study consists of young adults aged 18 to 34 years affiliated with La Trobe University. Participants will be recruited from La Trobe University (Bundoora and Bendigo campuses) through advertisement posters and email invitations.

### Inclusion Criteria

Participants will be eligible for inclusion in the study if they meet the following criteria: (1) adults aged 18 to 34 years; (2) ownership of a computer or smartphone with stable internet connectivity; (3) ability to read, write, and understand English; and (4) willingness and ability to provide informed consent.

### Exclusion Criteria

Participants will be excluded from the study if they meet any of the following criteria: (1) individuals with compulsive exercise behaviors or a history of disordered eating, as wellness content could be triggering; (2) inability to commit to a 3-month study duration; and (3) lack of basic digital literacy skills necessary to navigate web applications.

### Phase 3: Technical Implementation (Months 1-6)

#### Rationale for Technical Approach

PWAs represent an emerging approach to mHealth delivery, combining the accessibility of web-based platforms with native app–like functionality [45]. Unlike traditional native applications requiring platform-specific development, PWAs use standard web technologies to deliver cross-platform experiences [2]. For digital health research, PWAs offer (1) simplified deployment without the need for app store approval, (2) seamless updates without user intervention, (3) reduced development costs through single codebase maintenance, and (4) broader reach across devices and operating systems [45].

#### Technology Stack Selection

The front end uses HTML, CSS, and JavaScript modules. This decision prioritizes transparency and reproducibility of digital health interventions. The application uses a modular, component-based architecture, enabling separation of concerns across feature-specific files. Accessibility compliance follows Web Content Accessibility Guidelines (WCAG) 2.1 Level AA standards [46], implementing high-contrast color schemes (dark blue #1E3A8A and white #FFFFFF), dyslexia-friendly fonts (Open Sans, minimum 14 pt), and semantic HTML5 elements, operationalizing inclusive design principles [19]. The backend uses Firebase, and the empathetic chatbot sends requests to Google Dialogflow ES for natural language understanding. Firebase provides the data infrastructure through 4 integrated services: Firestore, authentication (email and password and Google OAuth 2.0), storage, and analytics (interaction tracking).

#### Development Methodology

Implementation follows the Agile software development methodology [20] with 12 two-week sprints conducted over 6 months, consistent with DSRM principles of iterative design and evaluation [21]. The lead researcher functions as both product owner and developer, maintaining close alignment between theoretical design specifications and technical implementation. Biweekly sprint reviews involve consultation with the research supervisor to validate theoretical fidelity across all frameworks, while periodic technical reviews with expert panel members (n=6) ensure adherence to accessibility and usability standards. Each sprint produces a deployable increment that is tested locally using Visual Studio (Microsoft Corporation).

The chatbot was initially configured with 50 predefined intents and corresponding responses. These responses were clinically verified against evidence-based sources, including the Diabetes Australia guidelines and the National Health and Medical Research Council recommendations, to ensure accuracy [17]. The key features of the MiCARE platform are summarized in Table 1.

**Table 1.** Mapping of design objectives to implemented features.

Design objective	Supporting theory	Implemented features
Empathy-driven interactions	SDT^a^ and CARE^b^	Empathetic chatbot with clinically verified responses and personalized reminders with supportive language
Equity-focused accessibility	Inclusive design and CARE	WCAG^c^ 2.1 AA compliance and multilingual interface capability at the architectural level, with English as the only language deployed and evaluated in this study
Culturally sensitive personalization	SDT and inclusive design	Symbolic visuals and culturally aligned responses (eg, halal meal suggestions)
Incremental goal setting	SDT	Dynamic goal-setting module, gamification (points and badges), and visual progress tracking
Intuitive onboarding	UCD^d^ and UTAUT^e^	Guided video tour, learning hub, and minimalist dashboard with grid-based tiles

a SDT: self-determination theory.

b CARE: compassion, assistance, respect, and empathy.

c WCAG: Web Content Accessibility Guidelines.

d UCD: User-Centered Design.

e UTAUT: unified theory of acceptance and use of technology.

#### Core Feature Implementation

A total of 6 core features operationalize the multitheoretical MiCARE design objectives derived from phases 1 and 2.

##### Feature 1: Empathetic Chatbot

The chatbot integrates Google Dialogflow ES, operationalizing empathy-driven interactions grounded in the CARE framework [17]. User messages are sent from the front end (chatbot.js) to the Node.js backend (server.js), which forwards requests to Dialogflow ES and returns responses. Dialogflow ES is configured with 50 initial intents covering diabetes prevention topics, with fulfillment responses crafted to follow CARE framework principles (ie, compassion, assistance, respect, and empathy).

##### Feature 2: Learning Hub

The learning hub uses modular content delivery, where educational snippets are stored as JavaScript objects and dynamically rendered based on user authentication state, supporting SDT’s competence construct through accessible evidence-based information [16]. Content was developed through a synthesis of Australian Dietary Guidelines (Diabetes Australia, 2024) and National Health and Medical Research Council physical activity recommendations, ensuring cultural relevance consistent with inclusive design principles [19]. Each snippet is concise (50‐100 words) to align with user preferences for minimal clutter identified during formative research and UCD principles [18].

##### Feature 3: Dynamic Goal-Setting Module

The user-defined goal-setting module implements through Firestore subcollections, operationalizing SDT’s autonomy and competence constructs [16]. Goals include attributes for type, target, progress, time stamps, and reset date.

##### Feature 4: Gamification System

The gamification system tracks points and badges stored in Firestore within each user’s rewards subcollection, supporting SDT’s competence and autonomy constructs through achievement recognition [16]. Points and badges are awarded automatically upon goal completion.

##### Feature 5: Personalized Reminders

Reminders are implemented as dismissible notification cards displayed contextually within the dashboard interface, preserving SDT’s autonomy construct through full user control [16].

##### Feature 6: Progress Dashboard

The dashboard aggregates data from multiple Firestore subcollections (rewards, goals, and interactions) and presents summary statistics through tile-based layout aligned with user preferences identified during the UCD co-design process [18]. Real-time synchronization ensures displayed metrics reflect the current state without requiring manual refresh through immediate feedback.

### Data From Phase 3

Technical implementation data will be documented through (1) sprint logs that record implementation decisions and rationale, (2) a clinical verification report for chatbot responses, (3) expert panel feedback on accessibility compliance, and (4) development timeline.

### Phase 4: Formative Usability and Feasibility Evaluation (Months 7-9)

Following completion of phase 3, a systematic formative usability and feasibility evaluation will be conducted with young adults (n=20) over a 3-month period. The evaluation uses the TTF [22] and UTAUT [23] frameworks as descriptive and exploratory organizing lenses.

### Recruitment

A total of 20 young adults aged 18 to 34 years will be recruited from La Trobe University (covering both rural and urban areas) through advertisement posters placed in common areas and follow-up emails to those expressing initial interest. The recruitment time frame will span 3 months, allowing sufficient time to reach the target sample size and follow up with potential participants. Sample sizes are informed by established usability testing guidelines [42,47] and qualitative saturation guidelines [43], which suggest that 15 to 20 participants can identify approximately 90% of usability issues. If the recruitment target is not achieved, the recruitment period may be extended.

### Data Collection

An online screening survey (10‐15 min) administered via QuestionPro (a platform licensed by La Trobe University) will determine eligibility. Potential participants will receive an email invitation with a link to the survey, accessible on various devices. Survey responses will be reviewed by authorized research personnel to confirm eligibility. Eligible participants will complete an initial survey (30‐45 min) collecting baseline data on familiarity with digital applications, current use patterns of similar applications, and expectations for the web application interface. This will be followed by accessing the web application via a provided link, with guidance from the research team if needed. Participants will be assigned a unique ID code for logging into the web application instead of using email or personal information.

Over the 3-month study period, participants will regularly interact with the web application’s interface and features, including navigating information pages, using the reminder system, using the goal-setting feature, and engaging with gamification elements. Weekly interaction is expected to be 15 to 30 minutes per week. These interactions will help evaluate usability and user satisfaction aspects of the design. Midway through the study (after 1.5 months), participants will complete a mid-study survey (30 min) providing feedback on their experience with the interface and specific design elements. At the end of the study, participants will complete a final survey (30 min) evaluating overall user experience and satisfaction with the web application design.

Real-time use analytics will be extracted from Firebase, including retention rates, feature interactions, navigation patterns, and session duration. These data will be analyzed descriptively to contextualize self-reported findings. Over the 3-month period, participants are expected to dedicate approximately 6 to 9 hours in total.

### Survey Instruments

Survey instruments have been developed based on the TTF [22] and UTAUT [23] constructs, assessing usability, usefulness, and satisfaction.

#### Usability

Usability is assessed using the following aspects: ease of navigation, interface clarity and organization, learning curve and intuitiveness, effectiveness of accessibility features, and technical reliability.

#### Usefulness

Usefulness is assessed using the following aspects: relevance of content and features, alignment with wellness goals, perceived value of personalized feedback, helpfulness of chatbot interactions, and effectiveness of goal-setting tools.

#### Satisfaction

Satisfaction is assessed using the following aspects: overall satisfaction with interface design, visual appeal and aesthetic preferences, emotional response to interactions, likelihood of continued use, and likelihood of recommending the platform to others.

#### Quantitative and Qualitative Measures

Surveys use both quantitative scales (1‐10 rating scales and Likert scales) and qualitative open-ended questions that allow participants to share detailed feedback, insights, and suggestions about their experience with the web application interface and design elements.

### Evaluation Frameworks

The TTF [22] and UTAUT [23] are used as organizing frameworks to structure data collection and interpretation.

The TTF [22] assesses the degree to which technology assists individuals in performing their tasks. For MiCARE, TTF evaluation examines the alignment between young adults’ wellness self-management needs and the platform’s features. Key constructs include task characteristics (eg, goal setting and information seeking), technology characteristics (eg, feature functionality and interface design), fit between tasks and technology, and performance impacts (eg, ease of wellness management).

The UTAUT [23] identifies factors influencing technology adoption. For MiCARE, UTAUT evaluation examines performance expectancy (perceived usefulness), effort expectancy (ease of use), social influence (peer recommendations), facilitating conditions (technical support and accessibility), and behavioral intention (likelihood of continued use).

### A Priori Success Criteria and Decision Rules

To support transparent interpretation and inform progression decisions, the following a priori success thresholds are defined:

- Usability—mean usability scores of ≥7 out of 10 across navigation, intuitiveness, and reliability items
- Acceptability—≥70% of participants reporting satisfaction scores of ≥7 out of 10
- Feasibility—≥60% participant retention at 12 weeks
- Behavioral intention—mean intention-to-use score ≥7 out of 10

Failure to meet these thresholds will prompt targeted design refinements prior to further evaluation. Meeting these thresholds will justify progression to a larger, more diverse sample evaluation.

### Data Analysis

#### Quantitative Data

Descriptive statistics will be calculated using RStudio, including means, medians, percentages, and frequency distributions for usability, usefulness, satisfaction, and behavioral intention measures.

#### Qualitative Data

Open-ended survey responses will be analyzed using thematic analysis supported by NVivo software (Lumivero LLC). Initial codes will be deductively derived from the TTF and UTAUT constructs, with inductive codes added for emergent themes. At least 2 researchers will independently code a selection of transcripts to achieve consensus and ensure scientific rigor.

#### Use Analytics

Descriptive summaries of retention rates, feature engagement frequency, session duration, and navigation patterns will be calculated to contextualize survey findings.

#### Data Triangulation and Handling of Divergence

Triangulation will be conducted by comparing self-reported survey responses, qualitative feedback, and objective use analytics at the feature level. In cases of convergence, aligned findings will be interpreted as reinforcing evidence of usability or acceptability. In cases of divergence (eg, high self-reported satisfaction but low objective feature engagement), qualitative open-ended responses will be examined to identify explanatory factors such as perceived usefulness without habitual use, usability friction, contextual constraints, or novelty effects. Divergent findings will not be resolved statistically but will be explicitly reported and interpreted as indicators of design refinement needs, consistent with formative mixed methods evaluation principles.

#### Application of the TTF and UTAUT Frameworks

Given the small convenience sample, the study is not powered to conduct statistical validation, hypothesis testing, or structural modeling of TTF or UTAUT constructs. These frameworks will be applied descriptively and exploratorily only to qualitatively map user feedback, usability perceptions, and engagement experiences to established theoretical domains. No inferential testing of relationships between constructs will be undertaken.

### Outcomes

#### Primary Outcomes

The primary outcomes include the following measures: usability scores (ease of navigation, intuitiveness, and accessibility), usefulness ratings (relevance, perceived value, and feature helpfulness), and satisfaction levels (overall satisfaction, visual appeal, and likelihood of continued use).

#### Secondary Outcomes

The secondary outcomes include retention rates over 3 months, feature engagement patterns (chatbot use, goal setting, and badge achievements), technical issues encountered, barriers to and facilitators of sustained use, and suggestions for interface improvements.

### Ethical Considerations

#### Overview

Ethics approval was obtained from the La Trobe University Human Research Ethics Committee (HEC24507) prior to commencing the phase 4 evaluation. This study focuses on UX design evaluation, assessing usability, usefulness, and satisfaction of the app. Clinical health outcomes are explicitly excluded from this research scope, and no health-related outcomes are being investigated. Participants will receive detailed information about the study objectives, procedures, data collection methods, and their rights as research participants before enrollment. Written informed consent will be obtained from all participants prior to participation. Participation in the study is voluntary, and participants may withdraw at any time without penalty. All collected data will be deidentified and stored securely to protect participant privacy and confidentiality. Only authorized members of the research team will have access to the data. Participants will not receive financial compensation for participation in this study.

Participants will be fully informed about the nature of the research, the types of data collected, how they will be used, and their rights, including the right to withdraw from the study. Informed consent forms will be provided and signed by each participant.

#### Data Management

Personal identifiers will be replaced with unique participant codes to anonymize data. All digital data will be stored on secure, encrypted drives (Research DataSpace and the La Trobe University P: Drive), while any incidental nondigital data will be kept in locked cabinets in secure, access-controlled rooms (PS1 212A) at La Trobe University. Access to data will be limited to authorized research team members only. Data will be retained for 5 years after completion of the research and will then be securely destroyed, ensuring ongoing confidentiality and privacy.

#### Withdrawal

Participants may withdraw at any time until data are fully anonymized (expected 1 week after final survey submission). After this point, individual data cannot be identified or removed from the aggregated dataset.

### Dissemination

Findings will be disseminated through (1) peer-reviewed journal publications in digital health, human-computer interaction, and health informatics venues; (2) conference presentations at workshops focusing on digital health, user experience design, and preventive health; (3) summary reports provided to participants highlighting key user experience insights; and (4) recommendations shared with La Trobe University and the broader digital health community.

The research team comprises transdisciplinary specialists in digital health innovation, implementation science, software engineering, human-computer interaction, and health informatics.

## Results

The study is scheduled for 9 months between October 2025 and June 2026. Phase 3 (platform development) commenced in October 2025 and is currently ongoing, involving the implementation of 6 core application features. Phase 4 (formative usability testing) is scheduled to occur between April 2026 and June 2026 and will involve approximately 20 participants aged 18 to 34 years recruited from La Trobe University campuses. Data collection will include screening, baseline, midstudy, and final surveys, together with real-time interaction analytics captured through Firebase. Data analysis will involve descriptive statistical analysis of survey responses and thematic analysis of qualitative feedback. The first results are expected to be submitted for publication in early 2027.

## Discussion

### Anticipated Findings

This protocol documents the systematic technical implementation and formative usability evaluation of MiCARE, a theory-driven PWA designed to support wellness engagement among young adults. Through comprehensive specifications grounded in multiple theoretical frameworks—SDT [16], the CARE framework [48], UCD [18], inclusive design [19], DSRM [20,21], TTF [22] and UTAUT [23]—we demonstrate a replicable methodology for translating multitheoretical frameworks into functional digital health interventions and conducting early-stage usability assessment.

The implementation methodology prioritizes transparency through web technologies, replicability through standardized architectural patterns, and accessibility through WCAG compliance [46]. By documenting code-level design rationale alongside theoretical foundations, this work provides digital health researchers and developers with a practical template for translating engagement frameworks into deployable interventions.

### Significance and Innovation

Few published studies document the technical translation from design specifications to functional software [38]. This protocol addresses the gap between conceptual frameworks and deployed interventions. This protocol provides comprehensive documentation of the full implementation process, enabling future researchers to adapt or extend the methodology.

The integration of behavioral theory (SDT and CARE) with implementation frameworks (DSRM, UCD, and inclusive design) and evaluation models (TTF and UTAUT) demonstrates a systematic approach to digital health development. This aligns with calls for theory-driven development in digital health [36,37] and addresses the need for rigorous evaluation methodologies [49]. This work is positioned as a design and implementation protocol accompanied by formative usability evaluation.

The JavaScript approach offers methodological advantages for research contexts. Unlike framework-dependent implementations, our approach remains interpretable without specialized knowledge, facilitating future maintenance by diverse research teams. This transparency aligns with open science principles and reproducibility standards in digital health research [50]. The Firebase-based data architecture demonstrates the practical application of serverless backend patterns in digital health research, minimizing DevOps complexity while maintaining scalability for future deployment.

### Limitations

Several limitations constrain interpretation and generalizability. First, the phase 4 evaluation recruits only 20 participants from a single university (La Trobe), all of whom are English-speaking, limiting external validity. This convenience, single-site, student sample supports heuristic usability discovery only and does not permit generalizable claims about acceptability, adoption, effectiveness, equity, or cultural tailoring beyond this specific setting. Accordingly, the TTF and UTAUT frameworks are applied descriptively and exploratorily only, and the study is not powered to test or validate relationships between theoretical constructs. This limitation is appropriate and expected for formative usability testing. Second, the implementation has been tested only in local development environments without deployment to production infrastructure. The planned phase 4 usability evaluation will assess real-world performance under authentic use conditions. Third, the exclusion of non-English speakers from the study is a major limitation, reducing the applicability and usability of the digital platform by culturally and linguistically diverse groups. Although the system architecture is designed to support multilingual features, only the English-language interface is deployed and evaluated in this study.

### Future Directions

Future work will build on the MiCARE platform’s design and data from phases 3 and 4 by conducting larger-scale evaluation with more diverse samples. Emphasis will be placed on increasing user diversity by including culturally and linguistically diverse populations and individuals with varying levels of digital literacy, addressing key barriers to equitable access identified in digital divide research. Factors such as socioeconomic status, internet access, and device availability, which exacerbate the digital divide, will be assessed to optimize platform uptake and use across diverse populations. A mixed methods approach will be used, combining quantitative data with qualitative insights to evaluate implementation outcomes and user engagement. To enhance inclusivity, multilingual interface support (expanding beyond English) and simplified navigation for users with lower digital literacy will be integrated, leveraging the scalable Firebase architecture and WCAG 2.1 AA compliance established in phase 3. Recruitment will expand beyond La Trobe University to community-based settings across rural and urban Victoria, Australia, ensuring a more representative sample of young adults. Subsequent phases will incorporate powered hypothesis testing with prespecified behavioral end points and corresponding analysis plans. Findings will inform scalable deployment strategies and contribute to the evidence base for theory-driven digital health interventions, addressing the gap between conceptual frameworks and real-world implementation.

### Conclusions

This study contributes a transparent design and implementation protocol for developing and evaluating MiCARE, a theory-driven PWA designed to foster sustained wellness engagement among young adults. By integrating SDT, CARE, UCD, inclusive design, DSRM, TTF, and UTAUT frameworks, this protocol bridges the critical gap between conceptual design and deployable digital health interventions. The systematic implementation of 6 core features, including an empathetic chatbot, learning hub, user-defined goal setting, gamification, personalized reminders, and progress dashboard, alongside a formative mixed methods usability evaluation aims to assess usability, usefulness, and satisfaction among a convenience sample of university-affiliated young adults. This work demonstrates a multitheoretical, user-centered PWA specifically tailored for preventive health engagement, addressing chronic disease risks such as prediabetes through culturally sensitive and accessible digital tools through a formative evaluation.

## Abbreviations

CARE: compassion, assistance, respect, and empathy
DSRM: design science research methodology
mHealth: mobile health
PWA: progressive web application
SDT: self-determination theory
TTF: task-technology fit
UCD: user-centered design
UI: user interface
UTAUT: unified theory of acceptance and use of technology
UX: user experience
WCAG: Web Content Accessibility Guidelines
